# Oral health and use of dental services in early adulthood and changes from adolescence - Fit Futures, a longitudinal cohort study

**DOI:** 10.1186/s12903-025-06241-6

**Published:** 2025-05-30

**Authors:** Elin Hadler-Olsen, Therese Bondø, Liv Marit Bergli, Andreas Schmalfuss, Hege Nermo

**Affiliations:** 1 The Public Dental Health Service Competence Center of Northern Norway, Tromsø, Norway; 2 Department of Medical Biology, Faculty of Health Sciences, UiT The Arctic University of Norway, Tromsø, Norway; 3 Department of Clinical Dentistry, Faculty of Health Sciences, UiT The Arctic University of Norway, Tromsø, Norway

**Keywords:** Dental health, Caries, Dental service utilisation, Young adults, Longitudinal study

## Abstract

**Background:**

The journey from adolescence to adulthood brings major life changes that can impact oral health and the utilisation of dental health services. Nevertheless, few longitudinal studies have followed oral health during this transition. This study aimed to evaluate key oral health parameters at young adulthood and how they have progressed from adolescence and identify predictors of self-reported dental health by young adulthood.

**Methods:**

Data from the first and third Fit Futures (FF) studies that follow a cohort of Norwegians from the first year of upper secondary school (FF1 2010–2011, mean age 16.6 years) for ten years (FF3 2021–2022, mean age 26.9 years), were analysed. Participants answered questionnaires and underwent dental examinations. We included all FF3 participants younger than 32 years at FF3 examination (*n* = 698, 54.7% female), of whom 642 (92.0%) had also attended FF1 and 584 (83.7%) had data on dental health from both studies. Data were analysed with cross-tabulations, related-samples Wilcoxon Signed Rank Test and multivariate binominal logistic regressions.

**Results:**

At age 16–17 (FF1) 53.0% and at age 26–27 (FF3) 46.0% of participants rated their dental health as very good or good (*p* = 0.002). Over the same period, the mean number of decayed teeth increased from 0.8 (SD 1.6) at age 16–17 to 1.4 (SD 2.1, *p* < 0.001) at age 26–27, with the largest increase among those reporting poor dental health. The proportion having four or more decayed teeth was 5.3% at age 16–17 and 14.4% at age 26–27 (*p* < 0.001). The mean number of filled teeth increased from 3.6 (SD 3.3) at age 16–17 to 4.8 (SD 3.7) at age 26–27 (*p* < 0.001), with the largest increase among those reporting good dental health. At age 26–27, 56.2% of the participants reported regular dental visits at least every second year, whereas 24.7% reported no or acute visits only. The primary barriers to regular dental visits were financial (30.6%), lack of priority (28.8%) or no subjective need (26.0%). Respondents with infrequent dental visits due to dental anxiety or finances reported especially poor dental health. Reporting moderate or poor dental health as a young adult was associated with male gender, having moderate or poor general health, caries experience and oral hygiene indicators in both longitudinal and cross-sectional regression analyses. Infrequent or irregular dental visits as a young adult was also associated with reporting moderate or poor dental health.

**Conclusions:**

The transition from adolescence to adulthood appear to be a critical period for dental health. Interventions such as subsidised care may improve dental health for many but will not address everyone’s need.

**Clincal trial number:**

Not applicable

## Background

The transition from adolescence to young adulthood involves substantial life changes for most people [1]. Many young adults leave their parental homes, pursue higher education, or establish professional careers, often with low or irregular incomes. These changes can adversely affect their diet, meal regularity, and oral health habits, including tooth brushing and use of dental services, all of which can ultimately impact their oral health.

In Norway, dental services are, by law, free for children and adolescents up to the age of 18 and strongly subsidised for 19–24-year-olds [2]. These age groups receive regular recalls for screening and necessary treatment at public dental clinics, and key parameters including the number of decayed, filled, and missing teeth are reported to a national registry. Until 2022, dental services were, with few exceptions, paid out-of-pocket from the age of 21, and mostly provided by private practitioners. However, over the last two years, the Norwegian government has decided to extend the eligibility for subsidised dental care to age 24 [3], a change that has been strongly debated [4]. One of the arguments against prolonging the period for subsidised dental care is that young adults in Norway generally have good dental health, and so the money could rather be spent on other groups of the adult population in greater need of support [5]. Nevertheless, caries is cumulative in nature, and tooth restorations have a limited lifespan. Therefore, investing in young people’s oral health may reduce their over-all need for dental treatments throughout life and support a good quality of life. A major problem for decision makers is the paucity of data on the oral health of the adult population and how it evolves from adolescence to adulthood. Previous cross-sectional studies in Norway have found a mean number of decayed teeth (DT) between 1.4 and 1.7 in young adults [6, 7], low utilisation of dental health services, and a relatively high proportion rating their dental health as moderate or poor [6]. Although some population-based studies have reported data on dental health parameters of the Norwegian adult population, very few are longitudinal.

In the present study, we use data from Fit Futures (FF), a longitudinal study following a cohort of first-year upper secondary school students (mean age 16 years) for ten years. The study aims were to investigate: (1) key aspects of oral health among young adults (cross-sectional); (2) the nature and self-reported determinants of their dental service use; (3) changes in key oral health aspects during the transition from adolescence to young adulthood (longitudinal); and (4) the influences on self-reported dental health in young adulthood.

## Materials and methods

### Study design and population

**Fig. 1:**
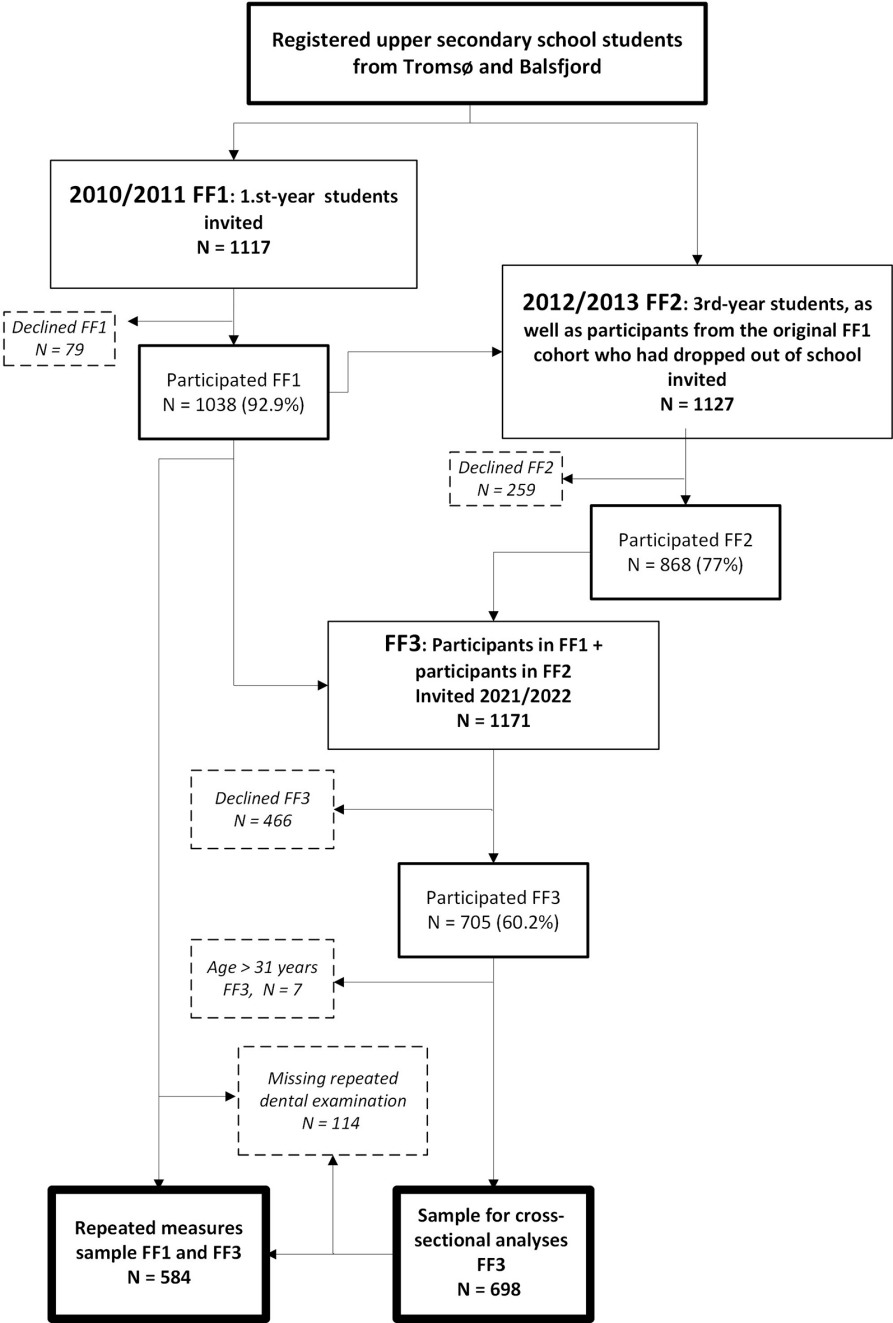
Flow of participants FF1 (age 16–17) to FF3 (age 26–27). This figure illustrates the flow of participants from Fit Futures (FF) 1 when participants were 16-17 years old to FF3 when participants were 26-27 years old. US-school: upper secondary school

This study uses cross-sectional data from the third FF wave (FF3) and longitudinal data from the first and third wave (FF1 and FF3) to assess key oral health measures among young adults and examine changes in these from adolescence to adulthood. FF1 was conducted in 2010–2011. All first-year students (age 16–17 years) attending upper secondary schools in two neighbouring municipalities in Northern Norway, Tromsø, an urban municipality and Balsfjord, a rural municipality, were invited (*n* = 1117). The participants (*n* = 1038, 93%) answered an extensive questionnaire and underwent clinical examinations as previously described in detail [8]. FF2 was conducted in 2012–2013, when the participants were in the third year of upper secondary school (age 18–19 years). The invitation to participate was extended to all students attending the third year of upper secondary school in Tromsø and Balsfjord municipalities, including those who did not participate in FF1, meaning that some of the FF2 participants had not taken part in FF1. FF2 did not include a clinical dental examination. In FF3 (2021–2022, age 26–27), everyone who had attended FF1 and/or FF2 were invited (*n* = 1171). At this time, many of the participants had moved away from Tromsø or Balsfjord and lived in various locations in Norway or abroad. All participants were offered reimbursement of their expenses for participation in the study that took place in Tromsø. The questionnaires and examinations in FF3 were essentially the same as in FF1, with the same procedures for recording caries and dental restorations. In total, 705 (60.2% of the invited) participated in FF3. Participants > 31 years in FF3 were excluded (*n* = 7) to have a more consistent group of participants, leaving 698 for cross-sectional analyses. Clinical dental data were missing for 49 of these participants as they either declined dental examination or because of a lack of capacity at the dental station due to the coronavirus pandemic. We have analysed the characteristics of FF1 participants who dropped out between FF1 and FF3. Of the 642 participants with data from both FF1 and FF3, we have clinical dental data from both FF1 and FF3 for 584 who constitute the longitudinal cohort. The flow of participants is illustrated in Fig. 1.

### Clinical oral examinations

A clinical oral examination was conducted in both FF1 (at age 16–17) and FF3 (at age 26–27). The procedures at age 16–17 have been described in detail previously [9, 10]. At age 26–27 the clinical oral examination included four bitewings and one panoramic radiograph, nine clinical intraoral photographs (Canon EOS 60D; Canon 105 mm with Sigma EM-140 DG Macro Flash Canon blitz, Tokyo, Japan), as well as a periodontal examination with registration of pocket depth and bleeding on probing. The pocket depth was measured to the closest millimetre from the gingival margin to the bottom of the pocket at four sites per tooth (buccal, disto-lingual, lingual, and mesio-lingual) using a North Carolina Probe (UNC15, LM1100-EV, Technomedics, Askim, Norway). Bleeding on probing (BOP) was registered at the same surfaces if bleeding occurred within about 20 s after pocket depth probing. The percentage of surfaces with BOP (BOP-index) was calculated for each participant. Third molars were excluded. One team with a dentist (TB) and a dental assistant (LMB) performed more than 90% of the examinations and the remaining were done by two back-up teams with dentists and assistants. Everyone involved in the clinical examinations were trained in all the procedures prior to the study.

### Variables

#### Health variables

**Decayed teeth (DT**,** at age 16–17 and at age 26–27):** The procedures for caries recording at age 16–17 (FF1) have been described in detail previously [9, 10]. At age 26–27 (FF3), one dentist (TB) did all the caries recording, which was based on radiographs and clinical, digital photographs. These were examined under standardised and optimal viewing conditions in a dark room. As in the first study wave, primary and secondary caries were recorded from radiographs using a 5-grade scale [11, 12]: (1) caries in the outer half of enamel; (2) caries in the inner half of enamel; (3) caries in the outer third of dentine; (4) caries in the middle third of dentine; and (5) caries in the inner third of dentine. For the evaluation of occlusal, buccal, and lingual caries the grading was aided by a picture illustration guide [13]. A tooth was denoted as decayed if one or more surfaces had caries grade 3–5 (dentine caries). Third molars were excluded from the analyses.

For cross-tabulations the number of DT was used as a continuous variable as well as categorised into: 0; 1; 2–3; and ≥ 4. In regression analysis DT at age 26–27 was used as a continuous variable, whereas DT at age 16–17 was categorised into: 0; 1; and ≥ 2 because the variable did not fulfil the assumption of a linear relationship with the log odds (criterion for logistic regressions) when used as a continuous variable.

**Filled teeth (FT at age 16–17 and at age 26–27)**: One examiner (TB) recorded tooth restorations based on radiographs and clinical photographs. Tooth surfaces restored with permanent fillings or permanent prosthetic restorations were considered filled, and a tooth was classified as filled if one or more surfaces were filled. Surfaces with temporary fillings were considered decayed, whereas surfaces with fissure sealants were considered sound. The number of FT was used as a continuous variable in analyses.

### Calibration and reliability

The calibration of caries recording at age 16–17 has been described previously [9]. In brief, the principal investigator was calibrated with two experienced dentists. For inter-rater agreement, the three examiners recorded caries on bitewing radiographs of 88 participants, giving a weighted Kappa value of 0.71 [9]. Prior to caries recording at age 26–27, the inter-observer agreement between the examiner and another experienced dentist was calculated for approximal and occlusal caries on molars and premolars on bitewing radiographs from six persons (288 surfaces). The intraclass correlation (ICC) between the two observers was 0.88 (95% Confidence interval (CI): 0.84, 0.90). Intra-observer agreement for caries recording was calculated for the primary examiner who recorded caries on ten participants (1280 surfaces) on two occasions with at least a two-month interval, giving an ICC of 0.84 (95% CI: 0.81, 0.87). Intra-observer agreement for the recording of FT was calculated by evaluating the radiographs and clinical photographs of 10 participants on two occasions with at least a two-month interval, giving a Cohen’s kappa of 0.86.

#### Variables from the questionnaires

**Self-reported dental health (SDH at age 16–17 and 26–27)**: Self-reported measures of global oral health are associated with clinical outcomes like caries, periodontitis, and tooth loss, as well as quality of life indicators [14–16], making them valuable indicators of overall oral health. This study used the Norwegian translation of Locker’s validated one-item measure [17]: “How do you rate your dental health on a 5-grade scale: 1) Very poor; 2) Poor; 3) Neither good nor poor; 4) Good; and 5) Very good?”. For longitudinal analyses the variable was used without recoding. For cross-tabulations we recategorised the answers into: (1) Poor (original options 1 and 2); (2) Moderate (original option 3); and (3) Good (original options 4 and 5). SDH at age 26–27 was used as an outcome in regression analyses. For these analyses, the variable was dichotomised into Poor or moderate dental health (original options 1–3) versus Good (original options 4 and 5).

**Toothbrushing frequency (at age 16–17 and 26–27)**: Participants were asked how often they usually brush their teeth. At age 16–17 the options were: (1) Less than once a week; (2) Once a week; (3) 2–3 times a week; (4) 4–6 times a week; (5) Once a day; and (6) Twice a day or more often. At age 26–27 the same question was asked, but with the options: (1) Less than once a week; (2) A few times a week; (3) Once a day; and (4) Twice a day or more often. For analyses the variables were dichotomised into: (1) Less than twice a day (original categories 1–5 at ag 16–17 and 1–3 at age 26–27); and (2) Twice a day or more often.

**Regular dental visits (at age 26–27)**: Participants were asked: ”Do you regularly visit a dentist or a dental hygienist?”, with the options: (1) Yes, more than once a year; (2) Yes, once a year; (3) Yes, every second year; (4) Yes, less than every second year; (5) No, only for acute problems; and (6) No, I never visit. For cross-tabulations options 5) and 6) were merged into acute or never. The variable was dichotomised into (1) At least every second year (original options 1, 2 and 3); and (2) Less than every second year (original options 4, 5 and 6) in the regression analyses.

**Reason for irregular dental visits (at age 26–27)**: Participants who reported having dental visits less than every second year (options 4–6 previous question) were asked to give the most important reason for not having more frequent visits, with the options: (1) I have not felt the need; (2) I have not prioritised it; (3) It is difficult to get an appointment; (4) I am anxious; (5) For financial reasons; and (6) Other reasons. Due to the limited number of participants answering this question, it was not included in regression analyses, only in cross-tabulations.

**General health (at age 16–17 and 26–27)**: Participants rated their general health on a 5-grade Likert scale from 1) Very poor to 5) Very good. For longitudinal analyses the variable was used without recoding. For cross-tabulations and regression analyses the answers were categorised into (1) Poor (original options 1 and 2); (2) Moderate (original option 3); and (3) Good (original options 4 and 5).

**Age and sex (at age 16–17 and 26–27)**: Information on age (birth year) and sex (man/woman) were from the questionnaires.

**Upper secondary school program (at age 16–17)**: Information was assessed in questionnaire (at age 16–17) with the options (1) Academic; (2) Sports; and (3) Vocational.

**Education (at ag 26–27)**: The participants were asked about the highest level of education they had completed, with the options (1) Primary school; (2) Occupational upper secondary school; (3) Upper secondary school; (4) College/University less than 4 years; and (5) College/university 4 years or more. In regression analysis the options were dichotomised into “No higher education (options 1, 2 and 3) and “Higher education” (options 4 and 5).

**Financial situation (at age 26–27)**: At age 26–27 the participants were asked about their family’s financial situation during their childhood and about their financial situation today, both questions had the following options: (1) Very difficult; (2) Difficult; (3) Average; (4) Good; and (5) Excellent. For cross-tabulation the variables were recategorised into (1) Difficult (original options 1 and 2); (2) Average (original option 3); and (3) Good (original option 4 and 5). In regression analyses the participant’s financial situation was dichotomised into (1) Difficult or average (original options 1–3); and (2) Good (original options 4 and 5).

### Statistical analyses

All analyses were conducted utilising the Statistical Package for Social Sciences (SPSS) version 26 (IMB corporation, Armonk, NY, USA), and the significance level was set at *p* < 0.05. In FF3 (at age 26–27), the inter- and intra-rater agreement was assessed by ICC (two-way mixed, absolute agreement, average measures) for scale variables (caries graded 1–5), and by Cohen’s kappa for categorical variables (FT). Key aspects of oral health, as well as the nature and self-reported determinants of dental service use were assessed with frequency analyses and cross-tabulations for the whole study cohort at age 26–27 and stratified on SDH at age 26–27. The statistical significance of the differences was determined using Chi-square tests for categorical variables and by independent samples Kruskal-Wallis tests for continuous variables. None of the continuous variables were normally distributed. They are presented as medians with interquartile range (IQR), except for the DT and FT variables which are also presented as means with standard deviation (SD) as this is the usual way of reporting these measures in studies [18], allowing comparison with other studies. Changes in key aspects of oral health during the transition from adolescence to young adulthood (longitudinal) were analysed with Related-samples Wilcoxon signed rank test.

To assess influences on SDH at age 26–27, binary logistic regression models were used. All regressions were run with forced entry and listwise deletion for missing data. We first had a model with independent variables at age 26–27 (cross-sectional). Reasons for no or infrequent regular dental visits were not included in the regression model as the question was only answered by a subgroup of the participants. The continuous variables fulfilled the assumption of a linear relationship between explanatory variables and the log odds. There was no multicollinearity between the explanatory variables (VIF < 1.5 for all variables included, no bivariate correlation coefficient (Spearman’s rho) > 0.30) or significant multiplicative interactions between sex and any of the other explanatory variables.

We had a separate regression model for longitudinal data to identify determinants of SDH during the transition from adolescence to young adulthood. The number of DT at age 16–17 did not fulfil the assumption of a linear relationship with the log odds when used as a continuous variable; therefore, it was categorised as described above. There was no multicollinearity between explanatory variables (VIF < 1.5, no bivariate correlation coefficient (Spearman’s rho) > 0.3) nor any multiplicative interactions between sex and any of the other explanatory variables.

Clinical dental variables were missing for 49 (7%) participants at age 26–27. For questionnaire variables data were missing for no more than 5% of the participants. Missing data were excluded from analyses.

## Results

### Characteristics of the cohort at age 26–27– cross-sectional data

FF3 included 698 participants, of whom 54.7% were women, with a mean age of 26.9 years (SD: 0.9, range 26–31 years) at the time of FF3 data collection. Table 1 presents SDH by participant characteristics at age 26–27 (cross-sectional data).

**Table 1:** Self-reported dental health at age 26–27 by demographic, socioeconomic and oral health characteristics

		Self-reported dental health at age 26–27			p*
Characteristics at age 26–27	All *N* (column %)	Good *n* (row %)	Moderate *n* (row %)	Poor *n* (row %)	
	698 (100)	304 (45.9)	283 (42.7)	75 (11.3)	
**Sex**					
Female	382 (54.7)	188 (51.5)	147 (40.3)	30 (8.2)	0.001
Male	316 (45.3)	116 (39.1)	136 (45.8)	45 (15.2)	
**Education**					
Secondary school	39 (5.9)	9 (23.1)	19 (48.7)	11 (28.2)	
US school academic	150 (22.6)	60 (40.0)	72 (48.0)	18 (12.0)	
US school vocational	107 (16.1)	37 (34.6)	47 (43.9)	23 (21.5)	< 0.001
Higher education < 4 years	191 (28.8)	98 (51.6)	78 (41.1)	14 (7.4)	
Higher education ≥ 4 years	176 (26.5)	100 (56.8)	67 (38.1)	9 (5.1)	
**Finances childhood**					
Difficult	59 (8.9)	23 (39.0)	25 (42.4)	11 (18.6)	
Average	212 (32.0)	91 (42.9)	90 (42.5)	31 (14.6)	0.053
Good	392 (59.1)	190 (48.6)	168 (43.0)	33 (8.4)	
**Finances today**					
Difficult	85 (12.8)	32 (37.6)	40 (47.1)	13 (15.3)	
Average	245 (37.0)	89 (36.5)	116 (47.5)	39 (16.0)	< 0.001
Good	333 (50.2)	183 (55.0)	127 (38.1)	23 (6.9)	
**General health**					
Good	481 (72.5)	259 (54.0)	189 (39.4)	32 (6.7)	
Moderate	140 (21.1)	35 (25.0)	75 (53.6)	30 (21.4)	< 0.001
Poor	42 (6.3)	10 (23.8)	19 (45.2)	13 (31.0)	
**DT**					
Mean (SD)	1.4 (2.1)	0.8 (1.3)	1.4 (1.9)	3.5 (3.1)	
Median (IQR)					< 0.001
**DT categories**					
DT = 0	328 (50.5)	176 (56.4)	121 (38.8)	15 (4.8)	
DT = 1	122 (18.8)	59 (50.0)	51 (43.2)	8 (6.8)	
DT = 2–3	110 (16.9)	37 (35.6)	53 (51.0)	14 (13.5)	< 0.001
DT ≥ 4	89 (13.7)	17 (20.5)	36 (43.4)	30 (36.1)	
**FT**					
Mean (SD)	4.8 (3.7)	4.1 (3.7)	5.0 (3.6)	6.4 (3.8)	
Median (IQR)					< 0.001
**Toothbrushing**					
≥2/day	477 (71.9)	253 (53.2)	191 (40.1)	32 (6.7)	
<2/day	186 (28.1)	51 (27.4)	92 (49.5)	43 (23.1)	< 0.001
**BOP-index** median (IQR)	24.1 (17.0)	21.4 (15.8)	26.8 (16.3)	30.4 (19.6)	< 0.001
**Dental visits**					
> 1/year	35 (5.3)	19 (54.3)	11 (31.4)	5 (14.3)	
1/year	202 (30.5)	124 (61.4)	65 (32.2)	13 (6.4)	
1/2nd year	135 (20.4)	71 (52.6)	52 (38.5)	12 (8.9)	< 0.001
<1/2nd year	127 (19.2)	51 (40.2)	66 (52.0)	10 (7.9)	
Never/acute	164 (24.7)	39 (23.9)	89 (54.6)	35 (21.5)	
**Why irregular visits**					
Not felt need	75 (26.0)	40 (53.3)	33 (44.0)	2 (2.7)	
Not prioritized it	83 (28.8)	27 (32.9)	43 (52.4)	12 (14.6)	
Anxious	34 (11.8)	3 (8.8)	21 (61.8)	10 (29.4)	< 0.001
Financial reasons	88 (30.6)	14 (15.9)	54 (61.4)	20 (22.7)	
Other reason	8 (2.8)	5 (62.5)	2 (25.0)	1 (12.5)	

*The statistical significance of differences was determined using Chi-square tests for categorical variables and by independent samples Kruskal-Wallis tests for continuous variables. Abbreviations: DT: Decayed teeth; FT: Filled teeth; IQR: interquartile range

The proportion with good, moderate, or poor SDH at age 26–27 was 45.9%, 42.7%, and 11.3%, respectively. There were relatively high proportions with poor SDH among men, participants with secondary school or vocational upper secondary school as their highest level of education, those reporting an average or difficult financial situation today and those reporting poor general health (Table 1). The prevalence of dentine caries at age 26–27 was 49.5%. The mean number of DT was 1.4, and respondents with good SDH had fewer DT than those with poor SDH. The mean number of FT was 4.8, where participants with good SDH had fewer FT than those with poor SDH. Participants with poorer self-care habits had worse SDH.

At age 26–27, when the participants had not been entitled to free or subsidised dental services for at least 6 years, 56.2% reported regular visits at least every second year, whereas 24.7% reported that they never had dental visits or visited for acute problems only. Among those reporting regular dental visits at least every second year, the majority had good SDH (Table 1). Participants reporting dental visits less frequently than every second year were asked for the most important reason for not having more frequent visits. Financial reasons were most common, closely followed by lack of priority and no subjective need. No one reported difficulties getting an appointment as the most important reason (excluded from Table 1). Participants naming anxiety or finances as the most important reasons for infrequent visits had the highest proportions with poor SDH (Table 1).

Table 2 presents the reasons for irregular dental visits by participants characteristics.

**Table 2:** Reasons for infrequent dental visits at age 26–27 by participant characteristics

	Why irregular dental visits					
Characteristics at age 26–27	No subjective need	Priority	Anxiety	Finances	Other	
n (row percentage)	75 (26.0)	83 (28.8)	34 (11.8)	88 (30.6)	8 (2.8)	p*
**Sex** n (% row)						
Female	24 (17.4)	43 (31.2)	23 (16.7)	45 (32.6)	3 (2.2)	0.007
Male	51 (34.0)	40 (26.7)	11 (7.3)	43 (28.7)	5 (3.3)	
**Higer education** n (row %)						
No	34 (23.3)	33 (22.6)	16 (11.0)	59 (40.4)	4 (2.7)	0.006
Yes	41 (28.9)	50 (35.2)	18 (12.7)	29 (20.4)	4 (2.8)	
**Economy** n (row %)						
Good	39 (34.2)	45 (39.5)	16 (14.0)	12 (10.5)	2 (1.8)	< 0.001
Average	29 (23.6)	26 (21.1)	12 (9.8)	52 (42.3)	4 (3.3)	
Difficult	7 (13.7)	12 (23.5)	6 (11.8)	24 (47.1)	2 (3.9)	
**General health** n (row %)						
Good	63 (33.0)	66 (34.6)	18 (9.4)	39 (20.4)	5 (2.6)	< 0.001
Moderate	10 (13.2)	14 (18.4)	14 (18.4)	36 (47.4)	2 (2.6)	
Poor	2 (9.5)	3 (14.3)	2 (9.5)	13 (61.9)	1 (4.8)	
**DT** mean (SD)	1.13 (1.63)	1.50 (2.21)	2.52 (2.71)	2.08 (2.39)	2.00 (1.91)	0.015^a^
**FT** mean (SD)	3.43 (3.07)	4.21 (3.38)	6.07 (4.08)	4.74 (2.81)	5.00 (3.56)	0.006^b^
**Toothbrushing** n (row %)						
≥2/day	19 (17.8)	26 (24.3)	12 (11.2)	47 (43.9)	3 (2.8)	0.003
<2/day	56 (30.9)	57 (31.5)	22 (12.2)	41 (22.7)	5 (2.8)	
**BOP-index** median (IQR)	25.9 (14.6)	26.8 (19.6)	25.9 (12.3)	28.6 (20.8)	19.6 (8.9)	0.202

^a^ DT: significant differences: between no subjective need and anxiety (*p* = 0.005) and finances (*p* = 0.014) and between priority and anxiety (*p* = 0.027). ^b^ FT: significant differences: between no subjective need and anxiety (*p* = 0.001), and finances (*p* = 0.005) and between priority and anxiety (*p* = 0.024). * The statistical significance of the differences was determined using Chi-square tests for categorical variables and by independent samples Kruskal-Wallis tests for continuous variables. Abbreviations: DT: decayed teeth; FT: filled teeth; IQR: interquartile range

Finances were the most common reason for infrequent dental visits among respondents without higher education, those having an average or difficult financial situation, those with moderate or poor general health and those who brushed their teeth twice or more a day. Lack of priority was the most frequent reason among those with higher education, a good financial situation, and those reporting good general health. No subjective need was the most common reason among men and those brushing their teeth less often than twice a day. Participants reporting anxiety or finances as the primary reason for infrequent dental visits had more tooth decay and more filled teeth than those having no subjective need or lack of priority as their main reason (Table 2).

A regression model was developed to best explain differences in SDH at age 26–27 based on the variables recorded at that age (cross-sectional data). We used poor or moderate SDH versus good as the outcome, and included sex, education, financial situation, DT, FT, toothbrushing frequency, BOP-index, dental visiting pattern, and general health as covariates (Table 3).

**Table 3:** Logistic regression model for poor or moderate dental health at age 26–27

	Poor or moderate dental health at age 26–27 versus good	
Characteristics at age 26–27	Unadjusted OR (95% CI)	Adjusted OR (95% CI)
**Sex**		
Female	Ref	Ref
Male	1.66 (1.22, 2.26)	1.48 (1.01, 2.17)
**Education**		
No higher education	2.13 (1.54, 2.89)	1.02 (0.69, 1.51)
Higher education	Ref	Ref
**Financial situation**		
Good	Ref	Ref
Average/difficult	2.10 (1.54, 2.86)	1.27 (0.87, 1.85)
**DT**	1.35 (1.22, 1.50)	1.17 (1.05, 1.31)
**FT**	1.10 (1.05, 1.15)	1.11 (1.05, 1.16)
**Toothbrushing**		
≥ 2/day	Ref	Ref
< 2/day	3.03 (2.08, 4.34)	1.41 (0.90, 2.23)
**BOP-index**	1.04 (1.03, 1.05)	1.02 (1.01, 1.04)
**Regular dental visits**		
≥1/2. year	Ref	Ref
<1/2. year	2.00 (1.24,3.04)	1.82 (1.13, 2.91)
Acute only or never	4.31 (2.85, 6.52)	3.46 (2.14, 5.59)
**General health**		
Good	Ref	Ref
Moderate	3.52 (2.30, 5.37)	2.48 (1.53, 4.04)
Poor	3.75 (1.80, 7.80)	4.53 (1.85, 11.09)

Abbreviations: DT: Decayed teeth; FT: Filled teeth; BOP: Bleeding on probing

The model explained 29% of the variance (Nagelkerke R^2^), where the following variables were significantly associated with reporting poor or moderate versus good dental health: male gender, DT (OR 1.17 for each additional DT), FT (OR 1.11 for each additional FT), BOP-index (OR 1.02 with each percentage higher BOP-index), infrequent/irregular dental visiting pattern, and reporting moderate or poor general health (Table 3).

### Changes over time - longitudinal data

#### Attrition analyses

There was attrition of participants between the first and the last study wave (age 16–17 (FF1) and age 26–27 (FF3)), and crosstabulations on retained participants versus dropouts were performed (Table 4).

**Table 4:** Attrition analyses age 16–17 to age 26–27

Cohort characteristics at age 16–17	Participated at age 16–17 and 26–27 *n* (column %)	Participated only at age 16–17 (Dropouts) *n* (column %)	
	636 (61.6)	396 (38.4)	p*
**Sex**			
Female	338 (53.1)	164 (41.4)	< 0.001
Male	298 (46.9)	232 (58.6)	
**Upper secondary school program**			
Academic	287 (45.1)	103 (26.0)	
Sports	68 (10.7)	36 (9.1)	< 0.001
Vocational	281 (44.2)	257 (64.9)	
**General health**			
Good	458 (73.0)	275 (75.3)	
Moderate	129 (20.6)	89 (24.4)	0.438
Poor	40 (6.4)	19 (5.2)	
**Emotional distress**			
No/little	488 (79.2)	306 (82.5)	
Moderate/severe	128 (20.8)	65 (17.5)	0.211
**Dental health**			
Good	333 (53.4)	228 (59.8)	
Moderate	236 (37.8)	109 (28.6)	0.009
Poor	55 (8.8)	44 (11.5)	
**Toothbrushing frequency**			
≥2/day	406 (64.8)	234 (61.1)	0.242
<2/day	221 (35.2)	149 (38.9)	
**DT mean (SD)**	0.9 (1.7)	1.1 (1.7)	0.005
**FT mean (SD)**	3.7 (3.4)	3.9 (3.6)	0.328

*The statistical significance of the differences was determined using Chi-square tests for categorical variables and by independent samples Kruskal-Wallis tests for continuous variables. Abbreviations: DT: Decayed teeth; FT: Filled teeth

The participants who dropped out between age 16–17 and age 26–27 differed significantly from those who participated in both study waves in several aspects. The dropout rate was relatively high among men and among participants in vocational study programs at upper secondary school. Furthermore, dropouts had a relatively low percentage with good SDH and a significantly higher mean number of decayed teeth than those who participated in both study waves (Table 4).

For respondents attending both waves of the study, differences in key oral health variables at age 16–17 and at age 26–27 were analysed and they are presented in Table 5.

**Table 5:** General and oral health characteristics at age 16–17 and at age 26–27

	Age 16–17 *n* (column %)	Age 26–27 *n* (column %)	P*
**Dental health**			
Very good	40 (6.9)	34 (6.1)	
Good	266 (46.1)	221 (39.9)	0.002
Moderate	220 (38.1)	234 (42.2)	
Poor	46 (8.0)	59 (10.6)	
Very poor	5 (0.9)	6 (1.1)	
**General health**			
Very good	143 (24.8)	105 (18.9)	
Good	284 (49.2)	306 (55.1)	
Moderate	115 (19.9)	111 (20.0)	0.143
Poor	31 (5.4)	29 (5.2)	
Very poor	4 (0.7)	4 (0.7)	
**DT**			
Mean (SD)	0.8 (1.6)	1.4 (2.1)	
Median (25, 75 percentile)	0.0 (0.0, 1.0)	1.0 (0.0, 2.0)	< 0.001
**DT categories**			
0	366 (62.7)	289 (49.5)	
1	98 (16.8)	108 (18.5)	< 0.001
2–3	89 (15.2)	103 (17.6)	
≥4	31 (5.3)	84 (14.4)	
**FT**			
Mean (SD)	3.6 (3.3)	4.8 (3.7)	
Median (25, 75 percentile)	3.0 (1.0, 6.0)	4.0 (2.0, 7.0)	< 0.001
**Toothbrushing**			
≥2/day	378 (65.4)	402 (72.4)	
1/day	134 (23.2)	127 (22.9)	< 0.001
<1/day	66 (11.4)	26 (4.7)	

*Related-samples Wilcoxon Signed Rank Test. Abbreviations: DT: Decayed teeth; FT: Filled teeth

The proportion with very good or good SDH decreased from age 16–17 to age 26–27 (53.4–45.9%, respectively, *p* = 0.002, Table 5). Fewer reported very good and more reported good general health at age 26–27 than at age 16–17, whereas proportions reporting moderate, poor or very poor general health were unchanged. The mean number of DT increased from age 16–17 to age 26–27, wheras the proportion having no DT decreased (Table 5). Also, the proportion having four or more DT was almost three times higher at age 26–27 than at age 16–17. The mean number of FT also increased between the studies, whereas more respondents reported that they brushed their teeth at least twice a day at age 26–27 than at age 16–17.

**Fig. 2:**
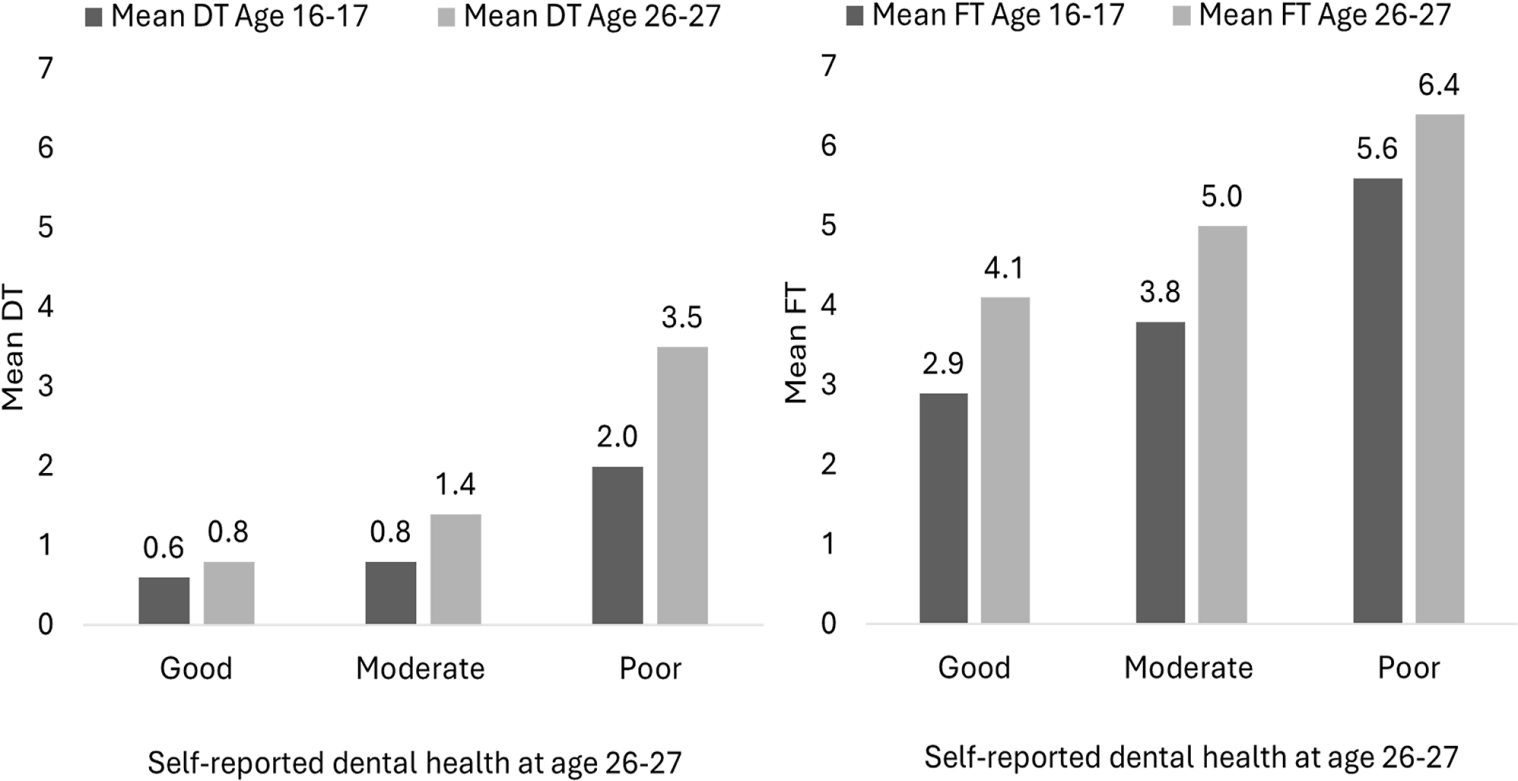
Decayed teeth and filled teeth at age 16–17 and age 26–27 by self-reported dental health at age 26–27. The figure shows the mean number of decayed teeth (left panel) and filled teeth (right panel) at age 16-17 and at age 26-27 by self-reported dental health at age 26-27

Figure 2 shows mean number of DT and FT at age 16–17 and at age 26–27 by SDH at age 26–27. Whereas the mean number of DT increased most among participants with moderate or poor SDH, the mean number of FT increased least among those with poor SDH (Fig. 2)

**Table 6:** Logistic regression model for poor or moderate dental health at age 26–27, predictors recorded at age 16–17

	Moderate or poor dental health vs. good	
Characteristics at age 16–17	Unadjusted OR (95% CI)	Adjusted OR (95% CI)
**Sex**		
Female	Ref	Ref
Male	1.66 (1.22, 2.26)	1.52 (1.06, 2.21)
**Upper secondary school program**		
Academic	Ref	Ref
Sports	1.79 (1.03, 3.10)	1.75 (0.97, 3.13)
Vocational	1.71 (1.21, 2.41)	1.20 (0.82, 1.76)
**General Health**		
Good	Ref	Ref
Moderate	2.17 (1.42, 3.32)	1.73 (1.09, 2.75)
Poor	3.87 (1.74, 8.64)	3.17 (1.38, 7.26)
**FT**	1.15 (1.09, 1.21)	1.11 (1.04, 1.18)
**DT**		
0	Ref	Ref
1	1.72 (1.09, 2.72)	1.37 (0.85, 2.24)
≥2	2.74 (1.77, 4.25)	1.59 (0.96, 2.63)
**Toothbrushing**		
<2/day	2.29 (1.61, 3.26)	1.74 (1.18, 2.57)
≥2/day	Ref	Ref

Abbreviations: Filled teeth; DT: Decayed teeth

A regression model was developed to find which variables recorded at age 16–17 significantly predicted SDH at age 26–27 (longitudinal data, Table 6). The model using moderate or poor SDH at age 26–27 versus good as outcome, and sex, upper secondary school study program, general health, toothbrushing frequency, number of FT, and DT (all recorded at age 16–17) as explanatory variables accounted for 16% of the variation in SDH (Nagelkerke R^2^). The variables that significantly predicted moderate or poor SDH at age 26–27 were sex (OR 1.52 for men versus women), general health (OR 1.73 for moderate versus good and OR 3.17 for poor versus good), number of filled teeth (OR 1.11 for each additional filled tooth), and toothbrushing frequency (OR 1.74 for less than twice a day versus twice or more often a day, Table 6)

## Discussion

In the present study, we assessed key oral health variables at young adulthood and how they had changed since adolescence in a cohort of young Norwegians. We also explored their dental visiting patterns and characteristics of low utilisers of dental health services. Finally, we determined variables that were associated with how the young adults experienced their dental health. We found that both clinical and self-reported oral health was impaired from adolescence to young adulthood

The overall prevalence of caries at age 26–27 was 49.5%, ranging from 44% among participants with good SDH to 95% among those with poor SDH. A recent meta-analysis found the mean prevalence of dentine caries to be 56% among European adolescents (12–19 years), with a somewhat lower prevalence in the Scandinavian region (49%) [19]. The FF cohort had a lower caries prevalence than reported in this meta-analysis at age 16–17 (38%), and about the same at age 26–27. In a previous study, we found a caries prevalence of 57% among 20-29-year-olds [6], which is higher than in the current study. At age 26–27 almost 14% of the participants had four or more DT, indicating a substantial need for treatment. In line with previous studies, we found that caries was indeed prevalent among young adults, and the notion that this part of the population has excellent dental health seems, sadly, an illusion

It is not surprising that health gradually declines with age, but the cohort followed in the present study were still in their twenties at the time of FF3, thus it is noteworthy that fewer than half of them had good SDH. The oral conditions responsible for most health loss are caries, periodontitis, and tooth loss [20]. Whereas severe periodontitis and tooth loss are rare among youth and young adults, caries is often prevalent in these age groups. Thus, it is reasonable to assume that this disease is an important cause of impaired SDH in the population under study. This was also supported by our findings that the odds of having moderate or poor SDH were greater by 17% for each additional DT, and by 11% for each additional FT recorded at age 26–27. The number of DT signifies active disease, whereas number of FT is a measure of the accumulated burden of disease, as well as an indirect indicator of access to dental health services. The mean number of DT increased more than the mean number of FT between age 16–17 and age 26–27. Whereas tooth decay increased most among participants with poor SDH, the increase in number of FT was greater among those with good or moderate SDH than among those with poor SDH. This suggests that dental health services may be less available to those with poor SDH. This is also supported by the relatively low utilisation of health services in the population under study, especially among those with poor SDH. A previous Swedish study following caries incidence from adolescence to young adulthood found that the incidence of caries decreased in young adulthood [21]. The apparent difference between that study and the present one may partly be explained by more frequent registrations in the Swedish study that allowed estimation of incidence rate. Due to the long interval between FF1 and FF3, we do not know how long the tooth decay at age 26–27 had been present. The low utilisation of dental services suggests that lesions may have been accumulated over a relatively long period in some participants, making it challenging to obtain valid incidence results in the current study. Also, the regular intervals between registrations in the Swedish study may have increased awareness of oral health among the participants and prompted positive dental health habits

Many studies have found that regular dental visits are associated with good oral health [22–25]. Although there is no consensus on how frequent these visits should be [26], 12–24 month intervals are common and supported by a previous study, reporting that rare visits were associated with higher odds of dental disease [27]. This is in line with findings in the present study showing a strong association between infrequent dental visits and having moderate or poor SDH in regression analyses. In the current study half of the respondents reported regular dental visits with 12–24-month intervals, and 25% reported that they had acute visits only or no visits at all. There was also a rather high proportion (19%) reporting regular visits with longer than 24-month intervals. This is a much lower utilisation of dental services than found in a previous study on a middle-aged and older population in the same area, where 83% had dental visits at least every second year [27], but slightly higher than reported in the 20–29-year subgroup in another cross-sectional population study [6]. In the current study, participants with longer than 24-month intervals between dental visits had poorer SDH than those with more frequent visits. However, the association with SDH varied significantly with the reason for the infrequent visits; participants who felt no need for more frequent dental visits had similar SDH to those with more frequent visits, whereas participants with infrequent visits due to dental anxiety or financial reasons had especially poor SDH and significantly more tooth decay. These findings are in line with previous studies [6], and suggest that a difficult financial situation and dental anxiety are strong barriers to seeking dental care. As finances were the most common reason for infrequent dental visits, prolonging the period for subsidised dental services may improve the accessibility of dental care for many of those in greatest need. To effectively reach individuals who avoid dental care due to anxiety, it is essential to recognise the current lack of systematic mapping and targeted interventions for dental anxiety within dental healthcare services. Addressing this gap will likely require alternative approaches that extend beyond standard care. This could include collaborating with mental health professionals and integrating evidence-based treatments, such as cognitive behavioural therapy, to better meet the needs of this vulnerable group. For people having infrequent dental visits due to a lack of subjective need or priority, subsidised dental services may be of little significance for their utilisation of dental services. Furthermore, more than half of the respondents who reported infrequent dental visits due to a perceived lack of need stated that they maintained good oral health and had fewer DT and FT than the mean of the study population. This suggests that individuals with minimal oral disease may successfully manage their oral health routines without frequent dental visits. However, this finding also underscores the importance of understanding the underlying reasons for not seeking dental care, which may be more significant than visit frequency alone

In accordance with previous findings among older adults [14], we found that reporting poor general health was strongly associated with having moderate or poor SDH in both cross-sectional and longitudinal analyses. The consistent association observed between SDH and self-reported general health may be due to shared risk factors between dental and other non-communicable diseases, reflecting overlapping constructs in self-reported measures related to health behaviours. It could also reflect a chain reaction of general diseases that affect diet, meal regularity, saliva production and composition, or the ability to clean teeth. These factors may have a direct impact on dental health. Furthermore, poor general health may impair the ability to work and, thereby, the financial situation, which in turn may affect dental visiting patterns and dental health. The findings emphasise the significant relationship between oral health and overall health and suggest a more interprofessional approach in dentistry, for example, by implementing preventive measures or increasing recall intervals for patients with underlying medical conditions while coordinating treatment with general practitioners. For participants reporting moderate or poor general health and low utilisation of dental services, finances were the most common reason for infrequent dental visits. Thus, subsidised dental services may improve the availability of dental care for this group. Since the link between oral and general health works both ways, improving oral health may lead to better overall health

None of the socioeconomic variables included in the regression models were significantly associated with SDH in adjusted models. Although not included in regression analyses, having financial reasons for infrequent dental visits was strongly associated with SDH in bivariate analyses, suggesting that economy may affect dental health through dental visiting pattern. A previous study following two Norwegian birth cohorts found that the social differences in oral health, assessed by the DMFS index, were reduced over a 33-year period from 1973 to 2006, but still existed [28]. Furthermore, other studies have found that differences in oral health by socio-economic status progressively increase as people age [29]. Thus, unless efforts are made to decrease differences in oral health in young adults, they may become larger over the participants’ life-course. In the present study, we found that participants with low socioeconomic status and poor dental health were likelier to drop out between FF1 and FF3. This suggests that certain demographic groups are not properly represented in our findings. Therefore, the impact of socioeconomic factors might be underestimated

In both longitudinal and cross-sectional analyses, men had a higher risk than women of having moderate or poor SDH as young adults. This corresponds well with findings from a recent review on sex differences in oral health, which found that compared to women, men more often ignore their oral health, have poorer oral hygiene habits, more irregular dental visits and have a higher prevalence of several oral diseases [30]. A possible explanation could be sex differences in health literacy. However, a recent national survey on health literacy in Norway found that women tended to have better health literacy than men, but the differences were insignificant [31]. Thus, there seems to be a need to increase our understanding of sex differences in oral health behaviour to promote oral health awareness and beneficial oral health habits more efficiently among men

### Study strengths and limitations

The study has several strengths and limitations. The longitudinal nature of the study allows us to explore potential predictors of dental health from adolescence to young adulthood, although direct causation cannot be determined. All measurements were performed according to research protocols by trained staff. We have subjective ratings of dental health and clinical measures of caries, fillings, and bleeding on probing. The outcome measure in the current study is self-reported. The cross-sectional regression showed significant associations between clinical dental health (such as caries experience) and SDH, which aligns with the current literature [17]. It should be noted that various factors beyond clinical findings can influence self-reported data. In general, self-reports tend to underestimate people at risk of developing diseases in terms of their overall health [32]. Individuals with higher socioeconomic status have been found to be more pessimistic when reflecting on their health, while those with lower socioeconomic status tend to evaluate their health optimistically. This optimistic evaluation may not reflect their actual risk for disease but rather uninformed beliefs about their health [33]. However, research has indicated that the global self-reported oral health measure can predict clinical tooth loss over time [15], making it a valuable outcome as it encompasses both clinical and subjective oral health experiences. The respondents were asked about the frequency of regular visits to a dentist or a dental hygienist, which we interpreted as regular check-ups. However, some respondents may also have included visits that were not for regular check-ups in their responses. The regression models explained 29% (cross-sectional) and 16% (longitudinal) of the variation in SDH as young adults (Nagelkerke R^2^). This means that important variables are missing from our models

The FF participants were recruited to the study during their first year of upper secondary school. Although not mandatory, most adolescents in Norway start upper secondary school, but the dropout rate is substantial. Therefore, the recruitment timing for the FF1 study provides a fair representation of the young population (age 16–17) in the region. However, the study population in FF3 (age 26–27) was subject to considerable attrition bias, which is evident from the dropout analysis. The participants in FF3 had a notably higher proportion of individuals who had completed at least upper secondary school (94%) than the national average for the 25-29-year age group in Norway (82%, according to Statistic Norway). Additionally, the percentage of individuals who had completed higher education, either shorter or longer, was slightly higher in FF3 (55% versus 51% (Statistics Norway)). These findings, coupled with the results of our attrition analysis, imply that the last wave (FF3, age 26–27) may not be representative of the general young adult population in Norway

## Conclusions

Our study demonstrates a marked decline in self-reported and clinical oral health from adolescence to young adulthood, suggesting that this transition is critical for oral health. Caries is cumulative in nature, and appropriate handling of the disease at a young age is important for the oral health in a life-course perspective. Our findings suggest that prolonging the period for subsidised dental care and addressing dental anxiety may reduce barriers to regular dental visits and, in turn, improve oral health for many young adults

## Data Availability

The datasets analyzed are available upon application to Fit Futures: fitfutures@uit.no.

## Abbreviations

FF: Fit futures
SDH: Self-reported dental health
DT: Decayed teeth
FT: Filled teeth
OR: Odds ratio
BOP: Bleeding on probing
CI: Confidence interval
SD: Standard deviation
IQR: Inter quartile range
